# Cupid’s quiver: Integrating sensory cues in rodent mating systems

**DOI:** 10.3389/fncir.2022.944895

**Published:** 2022-07-25

**Authors:** Nerissa E. G. Hoglen, Devanand S. Manoli

**Affiliations:** ^1^Department of Psychiatry and Behavioral Sciences, University of California, San Francisco, San Francisco, CA, United States; ^2^Center for Integrative Neuroscience, University of California, San Francisco, San Francisco, CA, United States; ^3^Weill Institute for Neurosciences, University of California, San Francisco, San Francisco, CA, United States; ^4^Kavli Institute for Fundamental Neuroscience, University of California, San Francisco, San Francisco, CA, United States; ^5^Neurosciences Graduate Program, University of California, San Francisco, San Francisco, CA, United States

**Keywords:** rodent, mating, social behavior, sensory processing, multisensory integration (MSI)

## Abstract

In many animal species, males and females exploit different mating strategies, display sex-typical behaviors, and use distinct systems to recognize ethologically relevant cues. Mate selection thus requires mutual recognition across diverse social interactions based on distinct sensory signals. These sex differences in courtship and mating behaviors correspond to differences in sensory systems and downstream neural substrates engaged to recognize and respond to courtship signals. In many rodents, males tend to rely heavily on volatile olfactory and pheromone cues, while females appear to be guided more by a combination of these chemosensory signals with acoustic cues in the form of ultrasonic vocalizations. The mechanisms by which chemical and acoustic cues are integrated to control behavior are understudied in mating but are known to be important in the control of maternal behaviors. Socially monogamous species constitute a behaviorally distinct group of rodents. In these species, anatomic differences between males and females outside the nervous system are less prominent than in species with non-monogamous mating systems, and both sexes engage in more symmetric social behaviors and form attachments. Nevertheless, despite the apparent similarities in behaviors displayed by monogamous males and females, the circuitry supporting social, mating, and attachment behaviors in these species is increasingly thought to differ between the sexes. Sex differences in sensory modalities most important for mate recognition in across species are of particular interest and present a wealth of questions yet to be answered. Here, we discuss how distinct sensory cues may be integrated to drive social and attachment behaviors in rodents, and the differing roles of specific sensory systems in eliciting displays of behavior by females or males.

## Introduction

Had I no eyes but ears, my ears would love. That inward beauty and invisible; Or were I deaf, thy outward parts would move each part in me that were but sensible: Though neither eyes nor ears, to hear nor see, yet should I be in love by touching thee.

Say, that the sense of feeling were bereft me, and that I could not see, nor hear, nor touch, and nothing but the very smell were left me, yet would my love to thee be still as much; for from the stillitory of thy face excelling comes breath perfum’d that breedeth love by smelling.

(William Shakespeare, *Venus and Adonis*)

Species’ survival requires mating; mating requires successful social interactions; successful social interactions require sensory signaling. The production and perception of the sensory cues that drive social interactions is one of the core questions in the study of animal behavior and its neural substrates. Nevertheless, as accurately and elegantly summarized over four centuries ago, no single sensory modality is necessary—or perhaps sufficient—to drive the displays that comprise social behaviors and attachment.

Rodents, especially laboratory mice, are among the principal model organisms in neuroscience due to their tractability and the proliferation of molecular and genetic tools for circuit dissection. The neural control of mating, including sensory processing of social cues, has been studied in rodents for decades. These foundational studies have made significant progress establishing nodes in a “social behavior network” for control of mating behavior as well as principles of sensory processing, but there is much yet to be determined to understand how mating is controlled in the rodent brain.

Social interactions are fundamentally multisensory processes, as multiple cues will co-occur over a range of timescales. A major area of open inquiry in the neuroscience of rodent mating behaviors is how information from multiple sensory modalities is integrated (multisensory integration, MSI) in the brain to provide an accurate representation of the identity and state of another individual, be they mate, rival, progeny, or predator. Rodents make use of a range of volatile and non-volatile chemical cues, somatosensation, audition, and vision in their interactions. While the processing of many of these cues has been established in a unisensory context, such stimuli rarely occur in isolation in naturalistic settings, opening many questions about the role these cues play in influencing the perception and downstream consequences of each other to elicit behavioral displays appropriate to distinct ethological contexts.

Multisensory integration has been established as a fundamental component of sensory processing, both by rigorous psychophysical and physiological studies that have focused on the initial mechanisms of sensory perception as well as the pathways that convey such information to the neural circuits that drive innate behaviors. The influence distinct sensory modalities exert over one another has been shown to be profound and is reflected in differences in neural activity in response to individual versus multiple cues in many loci throughout the brain. Mating interactions are rife with opportunities for multisensory integration, as conspecifics must detect potential mates, make decisions about whether or not to mate (perhaps choosing among several potential partners), respond appropriately to ongoing and reciprocal courtship behaviors, mate to successful fertilization, and, in monogamous species, encode the cues that identify a partner in order to form an enduring pair bond. These behaviors are all mediated by passing back and forth sensory cues across diverse modalities during varied social interactions.

The importance of combining modalities is becoming increasingly evident in studies of the neural control of mating behavior, but the mechanisms associated with integration are poorly understood. Furthermore, how such representations are encoded to drive attachment related behaviors—i.e., prosocial behaviors toward familiar individuals and rejection of novel potential mates—is even more obscure. Knowledge of the systems that control rodent mating behaviors, beginning with the unimodal sensory processing involved, affords access points to the circuitry of multisensory integration. Here, we review studies of rodent mating neural circuitry with an eye toward the overall role of sensory processing in mating and hypothesizing possible loci of integration in various forms.

## Sensory-guided male mating behaviors

The transfer of sensory information from one individual to another is the central goal of investigatory behaviors and a key component of the interactions that comprise courtship and mating behaviors. Investigatory behaviors in rodents include approach and tracking, sniffing of volatile and non-volatile (pheromone) odorants, whisking and grooming, audition of ultrasonic vocalizations, and vision of morphological and activity cues. These signals both drive and constitute ongoing, evolving components of sex-specific courtship and mating behaviors.

Male rodent courtship and mating behaviors include initial approach, chemosensory investigations, ultrasonic vocalizations, attempts to mount and intromit, and ejaculation. In this section, we consider how sensory stimulation via volatile and non-volatile olfactory, auditory, and somatosensory modalities affects male-specific courtship and mating behaviors.

### Volatile and non-volatile olfactory cues

Many decades of work examining innate behaviors and underlying neural circuitry in a range of rodent species has revealed a central and complex role for chemosensation in the control of male sexual behavior (for an elegant review and schematic, see Ishii and Touhara, 2019). A traditional model of two independent and parallel processing systems driving distinct behavior modules (Scalia and Winans, 1975; Halpern, 1987) is additionally influenced by the role of experience in controlling these behaviors. The two olfactory pathways for chemosensation are traditionally considered to operate largely in parallel, with the system originating in the main olfactory epithelium (MOE) thought to be responsive to volatile chemicals and the accessory olfactory system originating in the vomeronasal organ (VNO) responsive to non-volatile cues such as pheromones that are mechanically swept into the nasal cavity and bound by a variety of receptors in the aqueous mucosal environment. While males use both types of cues to evaluate the presence, health, and reproductive status of females, the accessory olfactory pathway has been shown to be important in sex discrimination and is thought to be responsible for innate behavior, ethologically appropriate displays in the absence of prior experience. In laboratory mice (*Mus musculus*), the VNO and AOB respond to female-specific urinary pheromones (Fu et al., 2015), and responses discriminate between male and female odors (Tolokh et al., 2013). Disrupting the function of the VNO by knocking out the gene coding for Trpc2 ion channels significantly abrogates odorant sensing in the VNO and results in indiscriminate male-typical sexual behaviors toward both sexes (and by both sexes as well) and eliminates displays of territorial aggression. Wildtype males typically attack intruder males while TRPC2 knockout males instead display courtship and mating behaviors (ultrasonic calling, mounting) usually directed only toward females (Stowers et al., 2002), suggesting that the presence of male pheromones suppresses mating behavior. Similarly, a lacrimal pheromone produced by juvenile laboratory mice, exocrine gland-secreting peptide 22 (ESP22; Ferrero et al., 2013) and pheromones generated during immune-response by female laboratory mice (Kwon et al., 2021) both inhibit mating behaviors in males, further implicating the accessory olfactory pathway as a curb on mating behaviors in inappropriate circumstances. In hamsters, however, surgical removal of the VNO of sexually naïve male hamsters abolished mating behavior entirely (Meredith, 1986), suggesting that mating behavior programs require activation by pheromones in this species. In male laboratory mice and prairie voles (*Microtus ochrogaster*), surgical ablation of the VNO similarly results in decreased—but not absent—mating behaviors (Clancy et al., 1984; Wysocki and Lepri, 1991; Wekesa and Lepri, 1994). The differences in these results across species indicate that the precise function of the VNO in male mating behaviors is not universal or singular across rodents. However, surgical ablations are not strictly comparable to genetic lesions that disrupt but do not eliminate VNO function, and such differences as well as species specificity may hint at a more sophisticated processing system that will be gradually uncovered as molecular genetic approaches are introduced into diverse species. Even with an incomplete understanding of species differences and the nuances of VNO function, it is clear that the accessory olfactory pathway plays a key role in regulating mating behavior in males.

The main olfactory pathway has been presented, in contrast, as mediating experience-dependent components of male sexual behavior. Male hamsters will mate with a novel female after ceasing mating with a familiar partner (Coolidge effect); this behavior requires intact main olfactory epithelium, but not VNO (Johnston and Rasmussen, 1984). Similarly, cues processed by the main olfactory system enable same-sex individual recognition after cohousing in spiny mice (*Acomys cahirinus*; Matochik, 1988); this phenomenon suggests a role for the main olfactory system in learning individuals’ identity broadly applicable across species and social situations. Individual recognition has long been associated with the major histocompatibility complex family of chemicals (MHC), which has been extensively studied in mice (reviewed in Brown and Eklund, 1994), as well as the major urinary proteins (MUPs; see discussion below). Across populations, MHC genotype drives disassortative mating (Yamazaki et al., 1976), suggesting a role for this system in mate choice in laboratory mice. Volatile chemicals as well as peptide compounds in urine have been shown to be key discriminable indicators of MHC genotype (Singer et al., 1997), supporting an important role for MOE and downstream processing of volatile odorants for individual recognition.

However, the roles of these two olfactory systems are more complicated and intertwined than this framing suggests. Early work on the role of the VNO in mate recognition and sexual behavior revealed that the VNO-dependence of many behaviors exists only in sexually naïve animals. Deficits in mounting and production of ultrasonic vocalizations (USVs, a key courtship behavior) after surgical VNO ablation are present only in sexually naïve male laboratory mice (Wysocki and Lepri, 1991) and hamsters (Meredith, 1986). Exposure to females also modulates expressions of aggression toward other males (Tan and Stowers, 2020). In prairie voles, which are socially monogamous and form pair bonds between mates, bonding status affects the ability of males to recognize individual females (Blocker and Ophir, 2015). Naïve, unbonded males are not able to discriminate among females in a habituation/dishabituation assay; male voles separated from the females by a barrier, preventing access to cues typically mediated by VNO, fail to discriminate familiar vs. novel females, indicating that individual recognition via non-pheromone cues develops as a consequence of bonding (or as a consequence of active detection of opposite-sex pheromone cues during the social interactions that facilitate pair bonding between mates). The experience dependence of VNO effects suggests that pheromone cues processed by the accessory olfactory system are integrated with other sensory cues, most likely coming from the main olfactory processing system, but potentially other modalities as well. Whether in the context of experience-dependent increases in agonistic displays or the dramatic changes in intersexual behaviors with novel potential mates following pair bonding, exposing males to female cues produces long-lasting plasticity in the brain that drives dramatic behavioral changes, reflecting asynchronous multimodal integration, as prior sensory experience affects responses to subsequent sensory cues.

It remains unclear precisely how the accessory pathway contributes to percept in inexperienced male rodents, and the specific mechanisms of the experience-dependent shift to using other cues are also unknown. It has been hypothesized that the VNO enhances the salience of MOE cues (Baum and Kelliher, 2009), but where and how this memory is formed remains an open question. Functional connections of the MOE to nuclei downstream of AOB that project back to AOB have been proposed, but this circuit has not been tested *in vivo* (Baum and Kelliher, 2009; Martel and Baum, 2009). Another instance of accessory olfactory processing sharing responsibility for behavioral outcomes is male hormonal responses to females. In laboratory mice and prairie voles, males experience a surge in luteinizing hormone that in turn stimulates testosterone after exposure to a female. This phenomenon can be triggered by anesthetized females and pheromones in isolation; both of those circumstances require intact VNO (Wysocki and Lepri, 1991). However, when the female is awake and behaving, the hormonal surge occurs even in the absence of functioning VNO, suggesting that other sensory cues can participate in driving this effect. The precise nature of these other inputs is unknown, as is whether multiple pathways are independently sufficient to produce the same effect, or whether there is an additive or superadditive effect of multiple modalities.

Both the main and accessory olfactory systems demonstrate some capacity to respond to the types of molecules typically associated with the other. The MOE responds to non-volatile MHC peptide ligands and is, unlike the VNO, necessary for males to demonstrate a preference for outgroup females based on these chemicals (Spehr et al., 2006). Furthermore, the accessory olfactory system also plays a role in individual recognition. While some indicators of MHC genotype are volatile, non-volatile MHC-associated peptides are also potent ligands in the VNO and may contribute to mate recognition in females (Leinders-Zufall et al., 2004). The accessory olfactory pathway also appears to mediate individual recognition via its responses to major urinary proteins (MUPs). Although the role of MUP sensing has been examined only in the context of male-male countermarking interactions in wild house mice (*Mus domesticus*; Hurst et al., 2001) and laboratory mice (Kaur et al., 2014), these observations demonstrate that individual recognition can be served by multiple pathways. Levels of MUPs expressed by females are dynamically regulated across the estrus cycle (Stopka et al., 2007; Černá et al., 2017), so non-volatile MUPs could plausibly contribute to male recognition of individual females. It is an open question how information from MHC and MUP signal repertoires, sensed by at least two olfactory modalities, is integrated to create complete individual profiles, but, as previously noted, such cues have the capacity to “combine to provide a highly polymorphic individual identity signal that is unlikely to be shared even among relatives” (Hurst et al., 2001). Even if MUP signaling plays no role in male recognition of individual females, information about sex sensed through the accessory olfactory system must be integrated with the volatile signals of individual identity to drive selective mating behavior, the mechanisms of which have not been directly tested.

### Ultrasonic and broad band vocalizations

While olfactory cues are a major mediator of sexual behavior in male rodents, a growing body of work implicates female vocalizations in courtship interactions in rodents, particularly laboratory mice. Early work in hamsters indicated that female hamsters produce USVs in response to male odorant cues, with the highest calling rates observed in estrous females (Floody et al., 1977), suggesting that female USVs may play a role in signaling sexual receptivity. Recently, this phenomenon has garnered attention in laboratory mice. While female laboratory mice vocalize at much lower rates than males, female USVs are associated with male USVs in temporal patterns that suggest interaction (Neunuebel et al., 2015). Furthermore, female USVs are associated with reduced female speed during male chases of the female, suggesting that, as in hamsters, female USVs may signal sexual receptivity and thus constitute a behaviorally relevant cue for males. Male responses to these calls are context-dependent; male laboratory mice adjust their movement speed to match female speed only during the initial stage of an interaction (Warren et al., 2020). Speed and assay time are indirect proxies for a complex range of social behaviors, but assessing speed requires integration of vocal responses with sensory information (most likely visual) about speed. The restriction of the behavioral effect to the first 10 min of interaction is likely to correspond to the period before mating, which further suggests an integration with sensory feedback about mating. Few other experiments have been conducted to test male responses to female USVs, but any effects are likely to be mediated in concert with chemosensory signals. Indeed, there is evidence of an “audience effect” on production of male USVs when in the presence of a female (Seagraves et al., 2016). Males increase production of USVs when both a female and other males are present. Notably, none of male urine, non-urinary odors, or playback of other male calls in the presence of a female recapitulates the changes to USV production observed in the presence of an awake behaving male, and even an anesthetized male produces only an attenuated effect. The insufficiency of any of the isolated signals to reproduce the effects of awake male suggests that multiple sensory inputs are required to be integrated to signal the presence of a male after female urinary cues are detected. Perhaps a combination of auditory and olfactory cues is needed to drive this behavior. How this relates to responses to female USVs remains to be seen.

Increased USV production in females may signal sexual receptivity while production of lower-frequency, broad band vocalizations (BBVs) is correlated with rejection behaviors (Finton et al., 2017). While males will persistently investigate BBVs emitted from a speaker when paired with female odorants (Grimsley et al., 2013), high BBV rates early in an interaction with an awake, behaving female predict a subsequent absence of mounting, whereas mounting rates are higher in interactions with lower BBV rates (Finton et al., 2017). It is unclear whether female BBVs motivate males to suppress mounting because calls often co-occur with aggressive rejection behaviors such as biting and kicking, as well as escape, but processing of these acoustic signals may be subject to heteromodal interactions with coincident olfactory and somatosensory cues.

### Combinations of cues

While volatile and non-volatile olfactory cues are sufficient in isolation for recognition of individuals in males of several rodent species, mating behaviors must be directed to a partner, and the male must orient correctly toward the female (the same is true of aggressive behaviors directed toward other males; Kaur et al., 2014). That olfactory-guided mating behaviors are displayed only when there is a partner present implies the integration of such olfactory cues with other sensory information that signals proximity and the orientation of another animal. Furthermore, access to a combination of olfactory, tactile, and visual cues prompts male laboratory mice to conduct more investigations of a juvenile stimulus mouse than any of these cues in isolation (Contestabile et al., 2021). While the mechanisms of this integration are largely unknown, a specific combination of olfactory cues and morphology, presumably processed by the visual or somatosensory systems, is necessary to drive pup-directed aggression in male laboratory mice (Isogai et al., 2018). Adult females may be recognized by a similar circuit process. Olfactory cues must also be integrated with some sensory indicator of mating to mediate mating-dependent behaviors across species. The Coolidge effect observed in hamsters, as well as the shifts from VNO to MOE mediated recognition cues in laboratory mice, necessitate a signal that mating has occurred. Similarly, male prairie voles must mate in order to form a pair bond (Insel et al., 1995). While the necessary signal of mating has not been specifically identified in any of these species, mating-specific induction of markers of neural activity is observed in the medial amygdala, a key relay for VNO-mediated chemosensory information, while modulation of activity in this region specifically disrupts the consummatory aspects of mating without affecting the preceding social and investigatory behaviors in Syrian hamsters (Newman et al., 1997) and rats (Huijgens et al., 2021). Thus, for example, somatosensory stimulation of the genitals or the neuroendocrine mediators released following ejaculation may signal mating (Bronson and Desjardins, 1982), as they appear to in the formation of olfactory memories in female prairie voles and laboratory mice (Williams et al., 1992; Otsuka et al., 2001).

## Sensory-guided female mating behaviors

Female rodent courtship and mating behaviors include hormonal regulation of estrus and pregnancy state, lordosis to allow mating access, and aggressive rejection of mating attempts. In this section, we consider how sensory stimulation in the olfactory, auditory, and somatosensory modalities affects these female-specific behaviors.

### Volatile and non-volatile olfactory cues

As with males, extensive study has revealed a key role of the two olfactory pathways in mediating female rodent mating behaviors (Asaba et al., 2014), prompting many of the same questions about the relative contributions and integration of these two pathways (see discussion above). However, the contributions of other sensory modalities are better understood in female rodents than in males, particularly the role of ultrasonic vocalizations (USVs). Nevertheless, many questions remain about how information from these two modalities is integrated to drive behavior (for an elegant review and schematic, see Asaba et al., 2014).

Olfactory cues mediated by both the main and accessory olfactory systems contribute to estrus induction, mate choice, and sexual receptivity or rejection in female rodents. Work in laboratory mice has shown that several odorants present in male urine stimulate approach and investigation in females, including the volatile chemical (methylthio)methanethiol (Lin et al., 2005) and the non-volatile atypical MUP darcin (Roberts et al., 2010). Other urinary chemicals allow female laboratory mice to distinguish information about the source male, including social status via farnesenes (Jemiolo et al., 1991), immune state (Kavaliers and Colwell, 1995a,b), androgen levels (Scott and Pfaff, 1970), and relatedness (Yamazaki et al., 1976), as well as discrimination of age by female meadow voles (*Microtus pennsylvanicus*; Ferkin, 1999). Work comparing lesions of the MOE and the VNO in laboratory mice indicates that both the main and accessory olfactory systems participate in driving indicators of receptivity such as lordosis, though MOE lesions only reduce these behaviors while VNO lesions abolish them entirely, and both systems can be used to distinguish individuals on the basis of sex and androgen levels (Keller et al., 2006a,b). Although recognition of individuals is thought to be largely mediated through volatile cues, typically from the MHC system, individual recognition of male laboratory mice by females can be mediated through non-volatile MHC-associated peptides (Leinders-Zufall et al., 2004) and non-volatile MUP combinations (Cheetham et al., 2007), consistent with the role of MUPs in mediating male recognition of other individual males (Kaur et al., 2014). Non-urinary odorants also contribute to female behavior; female mouse receptivity to male mounting attempts can be induced by the lacrimal pheromone exocrine gland-secreting peptide 1 (ESP1; Haga et al., 2010) and reduced by the juvenile lacrimal pheromone ESP22 (Osakada et al., 2018). Consistent with these findings from non-monogamous laboratory mice, in monogamous prairie voles, chemical cues mediated by the VNO are essential for both induction of estrus and formation of the pair bond (Curtis et al., 2001).

### Ultrasonic vocalizations

Vocalizations may play a similar role to odorants in driving investigation and receptivity and contributing to mate choice in female rodents. Males produce ultrasonic vocalizations (USVs) across a variety of rodent species, including laboratory mice (Pomerantz et al., 1983; Holy and Guo, 2005), Alston’s singing mice (*Scotinomys teguina*; Phelps et al., 2011), various species of hamsters including Djungarian hamsters (*Phodopus campbelli*; Floody et al., 1977; Pierce et al., 1989), prairie voles (Lepri et al., 1988), deer mice (*Peromyscus maniculatus bairdi*; Pomerantz and Clemens, 1981), Mongolian gerbils (*Meriones unguiculatus*; Holman, 1980), and montane voles (*Microtus montanus*; Pierce et al., 1989). Specific patterns of USV production during distinct stages of mating in different species suggest a variety of hypotheses about the precise function of USVs in courtship (Pierce et al., 1989); nuances of USV function are likely to differ across species. In laboratory mice, for example, the emission of USVs distinguishes putative mating-related mounts from aggression-related mounts, the latter of which represent dominance behavior that males direct toward other males without vocalizing (Karigo et al., 2021), suggesting that USVs may facilitate mating. Female responses to courtship USVs have been most thoroughly investigated in laboratory mice: call playback drives investigation, and acoustic features of calls may serve individual recognition (Musolf et al., 2010; Asaba et al., 2014; Chabout et al., 2015; Marconi et al., 2020). Wild-derived *Mus musculus* females prefer to investigate a speaker playing male USVs instead of a speaker simultaneously playing noise (Musolf et al., 2010). Reproductive success, as measured by subsequent birth of pups, is also higher for males with higher baseline USV production rates (Kanno and Kikusui, 2018), which suggests that USVs may play a role in stimulating female receptivity (without ruling out the possibility that USVs and fertility are both regulated by shared mechanisms). Indeed, socially dominant male laboratory mice produce USVs at higher rates (Wang et al., 2011), and males engaged in dominance behaviors (such as pursuing females or chasing other males) produce calls with distinct acoustic structure, after which the pursued females slow down (Sangiamo et al., 2020). Although the specific contributions of different sensory modalities to the assessment of dominance have not been tested, females prefer olfactory cues from socially dominant males (Jemiolo et al., 1991); USVs may thus provide a complementary, behaviorally relevant signal of social dominance.

Ultrasonic vocalization preference may also operate to drive outbreeding. Female laboratory mice preferentially investigate a speaker playing USVs from an unfamiliar male versus a speaker playing calls from a cohoused brother (Musolf et al., 2010) and prefer USVs from a male of an unfamiliar strain versus from a male of the strain of the adult male they were raised with (Asaba et al., 2014). Beyond kinship recognition, acoustic features of USVs may also signal vocalizer fitness, hormone status, and individual identity. Female laboratory mice preferentially approach playback of more spectrotemporally complex USVs over simpler ones, although both types of calls are typically produced during a mating interaction (Chabout et al., 2015). Similarly, female Alston’s singing mice preferentially spend time in proximity to speakers playing back faster examples of male vocalizations versus slower; interestingly, faster calls are associated with higher levels of androgens in males (Pasch et al., 2011). USVs produced by different individuals have differing acoustic properties (Liu et al., 2003). High interindividual and low intraindividual variation in call rate and repertoire size of male USVs in wild-derived *Mus musculus* may provide a substrate for individual recognition (Marconi et al., 2020), although females’ capacity to distinguish among individuals on the basis of USV signature has not been demonstrated.

### Combinations of cues

The information that may be conveyed by pheromones, volatile odors, USVs, and vaginocervical and trunk tactile sensations during mating invites questions about how these modalities are integrated. Are these information streams a redundant failsafe? Do congruent signals have an additive or superadditive effect? What happens in the case of conflicting information? While relatively few reports address these questions directly, there is some evidence that olfaction and audition may facilitate each other to drive mating behavior in female laboratory mice. In the absence of any other cues, female mice rapidly habituate to and stop preferentially approaching playback of male USVs (Hammerschmidt et al., 2009; Shepard and Liu, 2011). However, exposure to a male before re-exposure to playback reinstates female investigatory preference, indicating that exposure to male odorants or other cues may facilitate salience of calls on subsequent presentation (Shepard and Liu, 2011). Similarly, preference for USVs from outgroup males is dependent on prior exposure to male urinary cues (Asaba et al., 2014), suggesting a synergistic role between olfactory and acoustic cues in driving female behavior. Although the effects on females have not been directly studied, males also produce USVs during mating, not just during courtship, suggesting females may integrate USVs with tactile cues that occur during mating (Karigo et al., 2021). Further work is necessary to understand the full breadth of behavioral effects of the combination of these cues.

Following mating, female laboratory mice form a memory of mate identity (Kaba and Nakanishi, 1995). This memory is important for preventing termination of pregnancy by exposure to pheromones from the mate (Bruce effect), as can occur when females are exposed to urine from unfamiliar males. The physiological formation of this memory takes place via synaptic plasticity in the AOB and is triggered by vaginocervical stimulation (Keverne and de la Riva, 1982; Otsuka et al., 2001), revealing an integration between olfactory and somatosensory cues. The neural circuitry and synaptic mechanisms underlying this integration are well understood (see discussion below). Similarly, in female prairie voles, mating plays a role in pair bond formation. While females will show a partner preference (indicative of bonded status) after odor exposure in the absence of contact, mating decreases the length of exposure necessary from 24 h to 6 h (Williams et al., 1992). The neural mechanisms—including the specific sensory inputs—driving this phenomenon are yet unknown, but the process may be similar to the mechanism of olfactory and somatosensory integration observed in mice.

## Neural circuitry for integration of social signals

### Principles of multisensory integration

Utilizing multiple sensory modalities can increase the accuracy of an animal’s response to a salient event, decrease reaction time, expand the complexity of social information available, and form a unique multimodal percept, distinct from the component unisensory cues (Stein and Stanford, 2008). This combination of sensory information is termed multisensory integration (MSI), and the neural basis for integrating multiple senses has been studied extensively. Many initial studies were conducted in cats and primates, models in which vision, audition, and somatosensation drive sophisticated predatory and social behaviors, such as high-acuity gaze and pinna orienting in cats. The superior colliculus as well as sensory and association areas in cortex have all been identified as key loci for MSI at the level of single neurons. In these studies of MSI, stimuli are carefully controlled and typically synthesized by the experimenter to allow for evaluation of the relationship between specific psychophysical parameters and neuronal tuning-curves. This foundation led to the articulation of the concepts of subadditive, additive, and superadditive integration. An additive response describes the situation in which a neuron’s activity driven by combined stimuli is the sum of the responses produced by each stimulus independently; sub- and superadditive responses are when combined stimuli produce responses less than or greater than the sum of individual stimuli, respectively. Superadditive responses are expected when the individual stimuli are closer to the perceptual threshold and therefore harder to detect: a less robust stimulus gets a boost in discriminability from concurrent modulation by another modality. This phenomenon is known as the principle of inverse effectiveness. More recent studies in laboratory mice utilize the greater access of this model system to genetic manipulations and other tools, offering the promise of more thorough circuit dissection. However, to date, most of this work has focused on MSI of audition and vision (Siemann et al., 2015; Morrill and Hasenstaub, 2018; Knöpfel et al., 2019), or recently, modulation of somatosensation by audition (Zhang et al., 2020).

Multisensory integration is important for virtually any behavior that requires sensory input. Social behavior is no exception; successful social interactions require animals to correctly interpret cues with communicative significance across multiple sensory modalities. Our understanding of social interactions as responses to a rich tapestry of sensory information is expanding (Chen and Hong, 2018; Contestabile et al., 2021; Prior et al., 2022), as is our understanding of the way deficits in sensory processing and MSI play a role in human disorders of social behavior (Tseng et al., 2015; Siemann et al., 2020). Nevertheless, studies of MSI rarely make use of socially ethological stimuli or contexts and thus shed little light on the mechanisms of integration of concurrent cues relevant to social stimuli. Studies of the neural mechanisms of social behavior in rodents equally rarely make use of MSI theory to guide experiment design or evaluate results. Thus, there exists relatively little direct evidence of neural MSI in mating contexts in rodents. However, there are many candidate systems where integration of social cues in multiple modalities could be taking place.

### Multisensory integration by modulation of representations in sensory areas

Multisensory integration can take place downstream of primary sensory processing, in a population of neurons onto which inputs from two (or more) sensory processing streams converge. Heteromodal inputs can also directly modulate sensory representations, as is frequently the case in cortical sensory areas (Ghazanfar and Schroeder, 2006) and can also occur in non-cortical nuclei. There are sexual differences in olfactory (reviewed in Stowers and Logan, 2010) and auditory (reviewed in Lin et al., 2022) processing in laboratory mice and other rodents, as well as sex-specific experience-dependent plasticity in processing, such as changes to auditory processing during maternity (Marlin et al., 2015; Krishnan et al., 2017). However, sexual differences specific to MSI are less well understood, and further studies directly comparing male and female MSI responses are necessary. We review evidence for direct modulation of one sensory representation by another in sex-typical and mating-specific contexts in the following sections.

#### Proximity and orientation sensing

Many specific mating (as well as other social) behaviors require the presence of another animal to be expressed, suggesting that the indicator of presence or proximity is integrated with other sensory information. For example, male laboratory mice will attack male intruders to their territory. Males recognize other males via olfactory mediated cues; they respond with aggression when another male is present and countermarking when only the pheromone cue is present, indicating a role for presence-detection in pheromone guided aggression (Kaur et al., 2014). Similarly, pup-directed aggression is triggered by a combination of olfactory cues and specific morphological cues (Isogai et al., 2018). Multiple cues are also necessary for social behavior in non-aggressive contexts. Male mice increase female-directed USVs in the presence of other males; multiple sensory cues are necessary to drive this effect (Seagraves et al., 2016). Similarly, mice will stop avoiding a novel food if they smell or taste the food on another mouse, known as social transmission of food preference (STFP). Inducing STFP requires interaction with another animal; social odor alone is insufficient to induce STFP (Ryan et al., 2008). The exact sensory and circuit mechanisms driving detection of the immediate proximity of a conspecific are as yet unknown, but in mice, changes in locomotion after hearing a USV vary based on proximity to the USV emitter (Sangiamo et al., 2020) and approach behavior is affected by the locomotion of a social partner (Endo et al., 2018), indicating that perception of morphology (Isogai et al., 2018), proximity, and movement may play a role. Mice engage in nuanced orienting toward a social partner during investigation and mating, as well dominance and territorial interactions (Steinman et al., 2019; Duque-Wilckens et al., 2020; Williams et al., 2020), the exhibition of which is under genetic control (Defensor et al., 2011). This fine orienting may be based on visual information and tactile information relayed by trunk, paws, and whiskers. While the neural circuitry involved in these perceptions is relatively unknown, investigating a juvenile mouse drives more activity in the ventral tegmental area (VTA) than a toy with mouse odors (Contestabile et al., 2021), suggesting that the VTA may be involved in or downstream of such integration.

#### Tactile stimulation-dependent plasticity of pheromone responses

Following mating, female laboratory mice form a memory of the mate; this memory prevents exposure to the mate’s pheromones from inducing the Bruce effect. This memory involves experience-dependent plasticity in the female AOB following mating. Careful circuit dissection has demonstrated that tactile stimulation of the cervix is necessary for such chemosensory memory formation (Otsuka et al., 2001). This tactile signal stimulates release of noradrenaline in the AOB via a projection from the brainstem to AOB (Rosser and Keverne, 1985). Such noradrenergic input reduces mitral cell inhibition due to GABAergic signaling from granule cells (Kaba and Nakanishi, 1995) and decreases the paired-pulse depression that results from inhibition of mitral cells by granule cells, thereby increasing activity in the mitral cells contributing to memory formation (Otsuka et al., 2001). More recent work has used modern tools to further assess the contributions of different populations of cells in AOB to such encoding of olfactory memory. In contrast to the finding that mating increases activity in unspecified mitral cells in AOB, responsiveness in the mitral cells activated by mating is decreased following mating (Gao et al., 2017). The specific cell populations and nuanced changes to activity dynamics involved in this phenomenon hint at a sophisticated form of MSI to drive a process that is crucial to successful reproduction.

#### Modulation of vocalization responses

Olfactory and auditory cues co-occur in both mating and parenting contexts; the combination has been shown to have synergistic effects on behavior, which supports the proposal that integration of these cues must be occurring. Female mice demonstrate experience-dependent modulation of auditory cortical responses to pup calls by pup odors (Cohen et al., 2011), with pup odors increasing spontaneous and pup-call evoked spike rates in anesthetized mothers. Odor signals may reach auditory cortex via a basal amygdala projection that enhances pup call responses in maternal mice (Nowlan et al., 2022 preprint). The mechanism of maternal experience-induced plasticity is thought to involve a rebalancing of cortical inhibition (Marlin et al., 2015; Krishnan et al., 2017) via modulation by oxytocin (Oxt; Marlin et al., 2015). While these mechanisms have only been examined specifically in maternal circuitry, similar effects may be involved in integrating odorant and vocal cues involved in mating. Modulation of auditory responses may also occur earlier in the ascending auditory pathway prior to the auditory cortex: in male mice, serotonergic tone in inferior colliculus is inversely correlated with auditory and tactile rejection behaviors displayed by female partners, suggesting that auditory responses to female vocalizations or rejection may be affected by neuromodulation signaling affect at the level of the midbrain (Keesom and Hurley, 2016).

### Multisensory integration in nuclei of the social behavior network

Many areas across the rodent brain have been implicated in mating and other social behaviors. The specific neural mechanisms controlling these behaviors are often complex, involving many diverse populations of cells identified by their patterns of gene expression, anatomical location, functional and neuroanatomic connectivity, and patterns of activity across varied social contexts (for an elegant review and schematic, see Chen and Hong, 2018). While these populations often differ across males and females, many are also conserved across the sexes. Direct evidence for MSI is thin on the ground, but populations of neurons that have a role in behavior are observed to modulate activity in response to unimodal stimuli, offering promising candidates as loci of MSI, particularly when these responses are dynamically regulated by other factors including experience, hormones, or other behaviors. We provide a targeted survey of such regions in the following sections.

#### Medial amygdala

The medial amygdala (MeA) is a key relay in the processing of information from the VNO. It has been proposed that signals move from the VNO through the brain following a “labeled line” logic, whereby one signal passes with a direct mapping from one relay to another to eventually drive a specific behavioral response (Kimoto et al., 2007; Haga et al., 2010; Isogai et al., 2011). Indeed, some signals, such as the male pheromone ESP1, appear to control downstream activity and behavior in this way: ESP1 selectively drives responses of monosynaptically connected populations of neurons in a pathway from the AOB to the posteroventral MeA (MeApv), to the dorsal ventromedial hypothalamus (VMH), and finally, to the dorsal periaqueductal gray (PAG; Ishii et al., 2017). However, MeA representations of social information assembled from groups of socially relevant cues are also more complex and involve integration of multiple contexts and higher plasticity than the labeled line model suggests. Neural responses in the MeA of male and female mice can distinguish between conspecific and predator cues (Papes et al., 2010), and between male and female urine (Bergan et al., 2014) and whole animals (Li et al., 2017). These distinct responses are evident at both the single-cell and population level, and such population coding represents an important deviation from the labeled line model (Li et al., 2017). Additionally, the discriminability of MeA responses increases with sexual experience, as male mice show experience-dependent divergence of representations in MeA. While the mechanisms underlying such changes in discriminability are not completely understood, intriguingly, they require Oxt signaling in male but not female mice (Li et al., 2017; Yao et al., 2017), suggesting conserved mechanisms may underlie the encoding of social memories across diverse modalities and species in order to mediate varied patterns of social interactions and attachments (Insel, 2010).

While the dominant inputs to MeA are from the AOB (Bergan et al., 2014), principal cells in the MeApv also receive excitatory inputs from cells in the cortical amygdala (CoA; Keshavarzi et al., 2015), a key node downstream of the MOB. CoA inputs are spatially segregated on MeApv neurons. In keeping with the macrocircuit dominance of AOB inputs, at the single-cell level, CoA synapses provide less excitatory drive than those from AOB projections. While this observation provides a synaptic mechanism for convergent inputs from the main and olfactory systems in the MeA, the macrocircuit effects of this integration are unknown. In addition, a small population of MOB neurons appears to project directly to posterodorsal MeA (Kang et al., 2009). While these neurons have been shown via immediate early gene expression to respond to volatile urinary odors (Kang et al., 2009, 2011), their function *in vivo* is unknown. The mechanisms mediating the contributions of AOB and MOE information and their regulation of mating have been investigated at a genetic level (Mandiyan et al., 2005; Fraser and Shah, 2014; Matsuo et al., 2015). The integration of these pathways may contribute to learned associations between pheromones and volatile odors driving mate recognition, though the circuit substrates mediating such control remain uncharacterized. Many questions remain about the role of main olfactory inputs to the MeA, including what the significance of the anatomical separation of CoA projections from direct MOB projections to the MeA is, how either of these inputs contribute to integrated olfactory memories, and how they may contribute to transient or enduring modulation of pheromone responses.

#### Bed nucleus of the stria terminalis

The bed nucleus of the stria terminalis (BNST) has been implicated in rodent sexual behavior in studies of hamsters, gerbils, and laboratory mice. Along with the MeA, the BNST receives significant inputs from the AOB, indicating that it plays a role in processing pheromone cues. Studies using c-fos expression, a molecular proxy of sustained neural activity, identified neural responses in the BNST to opposite-sex odors and sexual experience in male golden hamsters (Fernandez-Fewell and Meredith, 1994), Syrian hamsters (Kollack and Newman, 1992; Wood and Newman, 1993), and gerbils (Heeb and Yahr, 1996) as well as in female Syrian hamsters (Joppa et al., 1995). The relative contributions of mating versus non-interactive odorant exposure are unclear; male golden hamsters with surgically ablated VNOs showed increased c-fos expression in medial BNST after mating, suggesting the effect may be independent of pheromone exposure, or—consistent with the non-specific, disinhibited mating behavior displayed by these animals—may reflect baseline activation in response to VNO-independent cues that elicit mating behaviors. Similarly, female Syrian hamsters show increased numbers of c-fos positive BNST neurons when interactions with a male include successful intromissions versus when intromissions are prevented (Ramos and DeBold, 2000), suggesting that activity in BNST may be driven by combinations of olfactory and tactile cues or reflect mating-related behaviors. Recent experiments utilizing fiber photometry for measurements of neural activity *in vivo* add evidence for the idea that the BNST responds to odorant as well as other cues to mediate mating behaviors. Recordings of Ca^2+^ fluctuations in the ventral BNST of male laboratory mice show that this neuronal population responds to both male and female odors, with larger magnitude Ca^2+^ fluctuations in response to female odors (Chen et al., 2020). A subset of these neurons defined by their projections to the premammillary nucleus accounts for the responses to male cues, suggesting that conspecific sex may be encoded by populations with distinct projections, although the observed responses to female odors are mediated by cells with unknown characteristics. Similarly, in the principle compartment of the BNST, the magnitude of population responses of aromatase-expressing neurons discriminates between male and female odors, with larger responses to female cues (Bayless et al., 2019). Notably, interaction with an awake, behaving animal—as opposed to isolated odor exposure—drives higher population responses, suggesting that pheromone cues may be integrated with other sensory information. Single unit recordings *in vivo*, a more thorough grasp of the circuitry and the specific patterns of connectivity of the heterogenous populations encompassed within these nuclei, and careful parsing of responses to different sensory cues are necessary to understand the role of BNST in integrating mating-related sensory cues across modalities.

#### Ventromedial hypothalamus

The ventromedial nucleus of the hypothalamus (VMH) has been implicated in mediating aggression and mating behavior in rodents for many decades, but a full picture of the complex genetic and functional divisions of this nucleus is still emerging. Experiments using electrical (Pfaff and Sakuma, 1979) and optogenetic (Lee et al., 2014) stimulation, optogenetic inhibition (Karigo et al., 2021), as well as genetic ablation (Yang et al., 2013), demonstrate a causal role for neurons in the ventrolateral portion of the VMH (VMHvl) in driving mating and aggressive behaviors in males (Lin et al., 2011); endogenous activity of neurons in this area increases during mating behaviors in both males (Lin et al., 2011; Karigo et al., 2021) and females (Hashikawa et al., 2017).

In addition to their role in controlling mating behaviors, VMHvl neurons may play a role in responding to cues from conspecifics (as opposed to the animal’s own behavior). The VMH receives inputs from MeA neurons carrying information about odorants (Choi et al., 2005; Carvalho et al., 2020). Projections from somatosensory cortex to the VMH have also been identified by tracing (Stanzani and Russo, 1980), but functional responses from these projections have not been recorded. While specific sensory inputs have yet to be thoroughly tested, convergence of inputs identified by tracing (Lo et al., 2019), inputs carrying information relevant to aggression (Krzywkowski et al., 2020), as well as dynamics of VMHvl activity suggest it is a locus of integration. Single-cell firing rates of VMHvl neurons in male mice briefly increase during investigation of a female, suggesting that the activity of these neurons may respond to sensory cues (Lin et al., 2011). However, these neurons are engaged in more than simple sensory representation, as they also respond during interactions with other males and exhibit different firing patterns under those conditions (Lin et al., 2011). The activity patterns of male-responsive VMHvl neurons expressing estrogen receptor 1 (Esr1+) cells grow more distinct from female-responsive cells with sexual and social experience (Remedios et al., 2017). These responses are generated during multimodal free interactions, but the experience-dependent change in population responses suggest that a signal of sexual experience is being integrated. Further work in this area is necessary to delineate unimodal responses and multisensory integration at the unicellular or population level.

The VMH also plays a role in controlling female receptivity and lordosis behaviors. Activity of VMHvl Esr1+ neurons in female mice increase activity during investigation of a male’s urine and during mating, but increase further during aggressive episodes, indicating that activity in this population does not constitute a simple unimodal representation (Hashikawa et al., 2017). Activity in both dorsal VMH (VMHd) and VMHvl has also been linked to lordosis, a stereotyped lumbar flexion associated with female receptivity in many rodent species. Originally characterized in rats (Pfaff and Lewis, 1974), tactile stimulation of the hindquarters induces lordosis robustly in this species. Hormone signaling across the estrus cycle as well as pheromones serve as permissive cues that facilitate lordosis behavior. The precise mechanisms of pheromone integration in neural populations remains to be demonstrated, but ESP1 facilitation of increased lordosis requires a population of neurons in VMHd expressing steroidogenic factor 1 (SF1; Ishii et al., 2017). Furthermore, work using fiber photometry has shown that neurons expressing Esr1 and progesterone receptor (PR) in VMHvl increase activity during lordosis and mating (Inoue et al., 2019; Liu et al., 2022), with smaller but still significant activity increases in response to male pheromone exposure (Liu et al., 2022). The activity of these neurons is necessary for lordosis, and their activity is modulated by hormonal state during the estrus cycle, positioning this population of cells as an important locus for integrating internal and—likely—sensory signals to control lordosis. RNA sequencing and chemogenic manipulations have further refined the identity of these cells, indicating that lordosis is specifically controlled by a cholecystokinin A receptor-expressing (Cckar+) subpopulation (Knoedler et al., 2022). In addition, a population of kisspeptin-expressing (kisspeptin+) neurons in the anteroventral periventricular area (AVPV) have been shown to be required for lordosis (Hellier et al., 2018). These neurons project to neuronal nitric oxide synthase-expressing (nNOS+) neurons in the VMH, and pharmacological manipulation of nNOS and kisspeptin signaling in VMH interferes with lordosis behavior, suggesting that kisspeptin signaling mediated by AVPV to VMH projections may be involved in lordosis (Bentefour and Bakker, 2021). Kisspeptin+ neurons are also activated by male pheromones and are necessary for the demonstration of mate choice (Bakker et al., 2010). Preferential locomotion toward attractive acoustic or olfactory cues may constitute a proximity-based integration of USVs, pheromones, or other chemosensory cues with lordosis-inducing tactile cues, but the involvement of neural populations in the VMH with lordosis behavior allows for the possibility of convergent sensory responses being integrated at the single-neuron or population level.

#### Medial preoptic area

Similar to the VMHvl, the medial preoptic area of the hypothalamus (MPOA) has a long history of being implicated in controlling mating as well as parenting behaviors (Stack et al., 2002), and recent work has greatly increased our understanding of the particular roles of specific populations of cells within this region (Wu et al., 2014; Kohl et al., 2018). In female mice, neurons in the MPOA expressing neurotensin (Nts+) show increased levels of activity when a mouse investigates male as opposed to female urine (McHenry et al., 2017), suggesting that these neurons play a role in sex discrimination or mating behaviors. This increase in activity is modulated by estrus cycle stage, and preference for males is impaired by optogenetic inhibition of these cells. Similarly, in male mice, population activity in the MPOA increases in response to presentation of a female, in particular a female that elicits mating behaviors, as larger peaks in activity are observed during investigations followed by a mounting attempt (Wei et al., 2018). It is unclear whether this activity differential reflects a threshold of activity necessary for mounting or whether the difference may represent integration with sensory feedback due to mounting. Optogenetic stimulation of these cells drives mounting in both males and females when in the presence of a target; interestingly, a toy mouse is insufficient to elicit mounting in combination with stimulation, in contrast to juvenile mice or rats under these conditions despite their inappropriate pheromones, suggesting yet-to-be-determined permissive sensory cues are required even with MPOA stimulation. Thus, it seems that activity of MPOA cells may be able to replace or override chemosensory signaling; how this activity is integrated with the morphological cues provided by a target is unclear. The role of MPOA in driving mounting is perhaps specific to Esr1+ cells (Karigo et al., 2021); optogenetic stimulation of this subpopulation of MPOA cells is sufficient to drive mounting attempts directed even toward inanimate objects. It remains possible that broader stimulation of populations outside these Esr1+ neurons recruits cells that enforce the requirement for a more plausible mount recipient or otherwise respond to cues that inhibit these behaviors in ethologically inappropriate contexts (Wei et al., 2018). Nevertheless, MPOA function is directly tied to mating behaviors in both male and female mice, but more work is necessary to determine what, if any, MSI occurs in this nucleus.

#### Prefrontal cortex

The prefrontal cortex (PFC) is a cortical region implicated in a wide range of behaviors across species. In laboratory mice, the PFC receives inputs from across sensory cortex (as well as a variety of other nuclei) and plays a role in many non-social behaviors (reviewed in Le Merre et al., 2021). The PFC is additionally thought to play a role in mediating decisions between participating in agonistic social versus non-social behaviors (Gangopadhyay et al., 2021) and in responding to novel social stimuli (Frost et al., 2021), as well as in attachment related behaviors (Amadei et al., 2017). Given its prominent role in learning and flexible behaviors, relatively little attention has been paid to potential contributions of PFC to mating behavior. However, the PFC has a significant role in integrating sensory information (e.g., Le Merre et al., 2018), and a few recent reports have also demonstrated a role for PFC in social recognition and mating behaviors. In male mice, single neurons and neuron populations in dorsomedial PFC (dmPFC) encode conspecific sex during free interactions, with more cells showing increased activity to females than to males (Kingsbury et al., 2020). This distinct neural response is specific to interacting with a social partner and is not recapitulated by investigating odors, suggesting that other sensory information is being used and integrated for this phenomenon or that this population of cells is downstream of sensory integration occurring elsewhere. In female mice, ablating a specific population of interneurons expressing both somatostatin (SST+) and oxytocin receptor (OxtR+) or pharmacologically blocking the action of Oxt reduced time spent investigating a male specifically during estrus, i.e., during sexual receptivity (Nakajima et al., 2014). While these studies do not address the question of unimodal responses to sensory cues, the effect demonstrates a role for integration of Oxt and steroid hormones in PFC. Interestingly, a distinct population of glutamatergic OxtR+ cells in PFC in male mice are involved in recognition of a familiar versus novel male (Tan et al., 2019). Same-sex recognition is not a mating behavior, but PFC sensory responses to the two sets of cues may be similar and parallel responses to novel opposite-sex mates vs. intrasexual dominance. When compared to several different non-social odors, neurons responding to urine from males and females (presented separately) together formed a group more distinctive from the grouped non-social odors than from each other, suggesting that PFC encodes the social status of odors rather than individual odor identity (Levy et al., 2019). While Oxt modulation of sensory responses in PFC has not been tested, in other cortical and subcortical areas, Oxt modulates sensory responses to social stimuli (Marlin et al., 2015; Yao et al., 2017).

#### Insula

Based on work in humans and other primates, the insular cortex (insula) is known to play a key role in integration of affective states and sensory cues relevant to social behavior (reviewed in Rogers-Carter and Christianson, 2019). Although no published experiments provide evidence of insula involvement in rodent mating behaviors, responses to non-mating social cues as well as robust MSI have been observed in mouse insula. Superadditive modulation of responses to pure tones by air puffs, following the principle of inverse effectiveness, has been observed in the insula of anesthetized mice (Gogolla et al., 2014), indicating that classical MSI occurs in this region. Furthermore, neurons in the insula of male mice are modulated by social interaction with another male (Miura et al., 2020). Nevertheless, the specific sensory inputs mediating this effect, as well as the involvement of the insula in mating behaviors, remain to be tested.

## Conclusion

Much work remains to be done to understand the role and mechanisms of multisensory integration in rodent mating behavior and how these representations and pathways contribute to the social memories that comprise social attachment. The diversity of both mating and social organization systems across rodents and other phyla implies that distinct cues that identify individuals, their reproductive state, and other measures of physiology and health are integrated to build and maintain systems of social structure via enduring relationships between individuals and groups. Monogamous pair bonds in particular require a robust system of recognizing individuals; while this increased need for reliable recognition is perhaps unlikely to drive dramatic differences in sensory processing capacities between monogamous and non-monogamous species, comparative neuroanatomical studies suggest there may be expansions in multisensory processing capacity in monogamous species (Campi et al., 2007; Kingsbury et al., 2012). Further studies that make use of direct species comparisons with consistent stimuli and behavior will help elucidate what mechanisms of sensory processing are specific to distinct social systems. Though outside the scope of this review, the representations of conspecifics generated through the integration of sensory cues described here must additionally incorporate hormonal and neuroendocrine information about an individual’s and others’ reproductive status and health, and—in the context of some mating systems—social status, cues whose identity and pathways for detection and processing remain to be determined. The function of neurons and circuits in these critical behaviors has implications for disorders of social behavior and sensory processing and the evolution of social behavior across mammals. The patterns of social interactions that facilitate mating and attachment evolve rapidly but appear to converge onto common molecular– and perhaps neural—substrates. Understanding these circuits in the rapidly expanding menagerie of model organisms, and how they are disrupted by genetic lesions or adverse experiences that contribute to disorders that impact social cognition and attachment, will illuminate the mechanisms that mediate these fascinating behaviors and how they may be targeted to improve health and wellness.

## Author contributions

NH and DM contributed to the conception and writing of this manuscript. Both authors contributed to the article and approved the submitted version.
